# Supplementary material for: Statistical Copolymers of n-Butyl Vinyl Ether and 2-Chloroethyl Vinyl Ether via Metallocene-Mediated Cationic Polymerization. A Scaffold for the Synthesis of Graft Copolymers
Source: Polymers (Basel). 2019 Sep 16;11(9):1510. doi: 10.3390/polym11091510 (PMC6780830; doi:10.3390/polym11091510)
Supplement: Supplementary file 1 [file polymers-11-01510-s001.pdf]

Statistical copolymers of n-butyl vinyl ether and 2-chloroethyl vinyl ether via metallocene mediated cationic polymerization. A scaffold for the synthesis of graft copolymers

*Stavros Zouganelis, Ioannis Choinopoulos, Ioannis Goulas and Marinos Pitsikalis**

Industrial Chemistry Laboratory, Department of Chemistry, National and Kapodistrian University of Athens, Panepistimiopolis Zografou, 15771 Athens Greece

Supporting Information

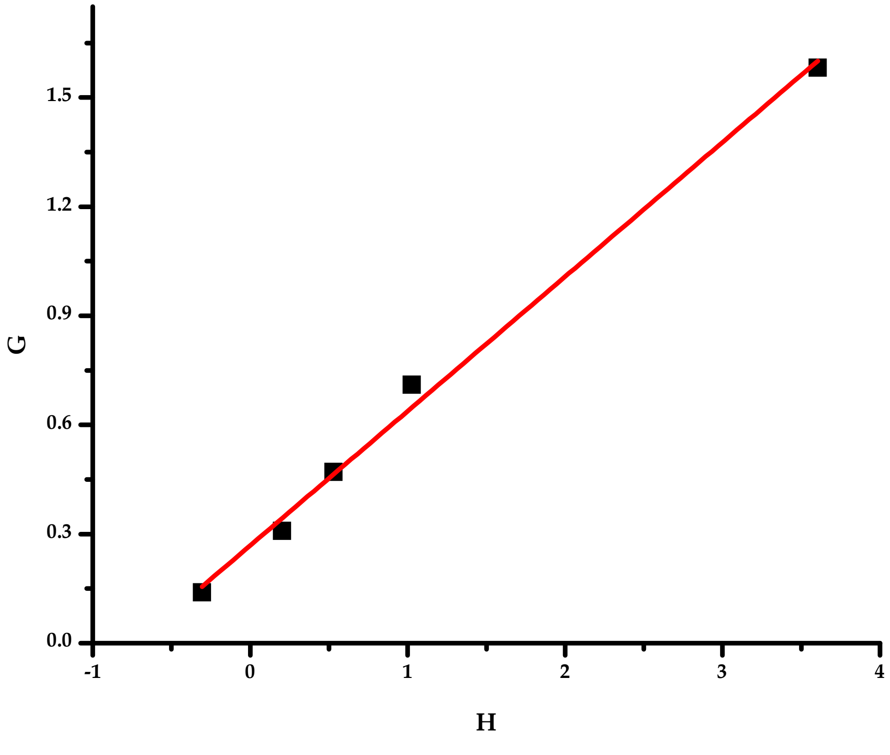

Figure S1. FR plot for the determination of the reactivity ratios for the PBVE-co-PCEVE

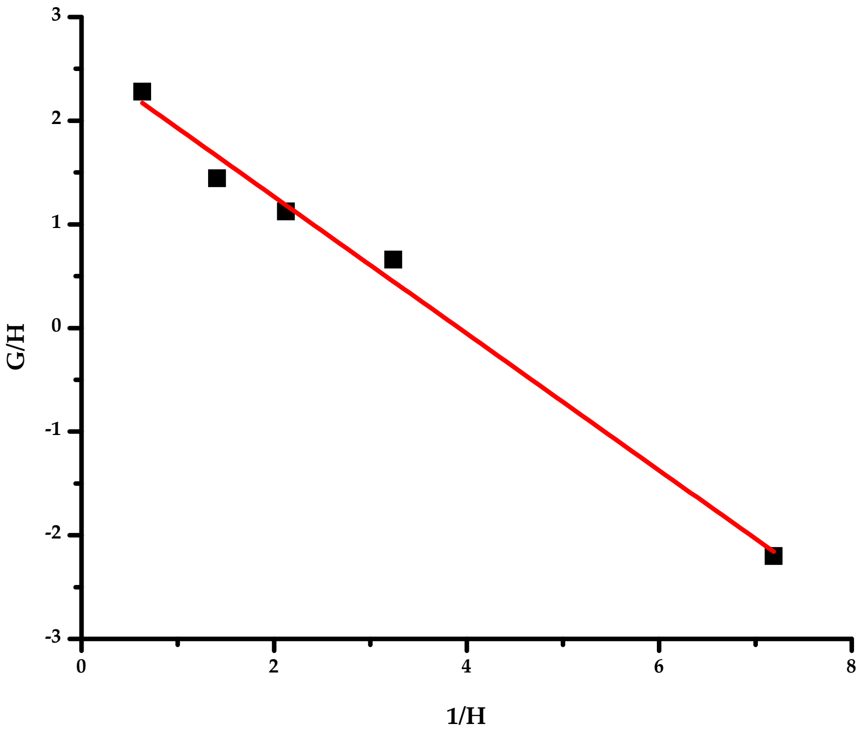

Figure S2. iFR plot for the determination of the reactivity ratios for the PBVE-co-PCEVE

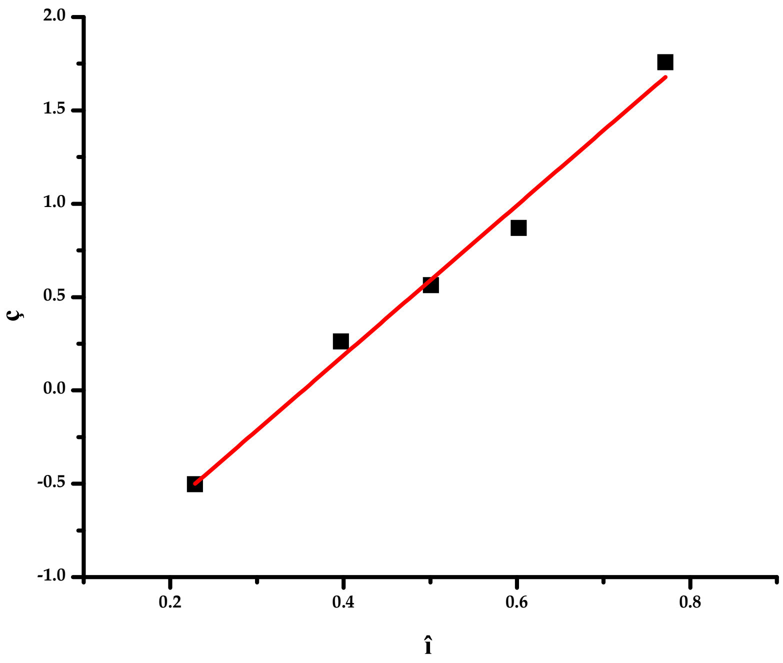

Figure S3. KT plot for the determination of the reactivity ratios for the PBVE-co-PCEVE

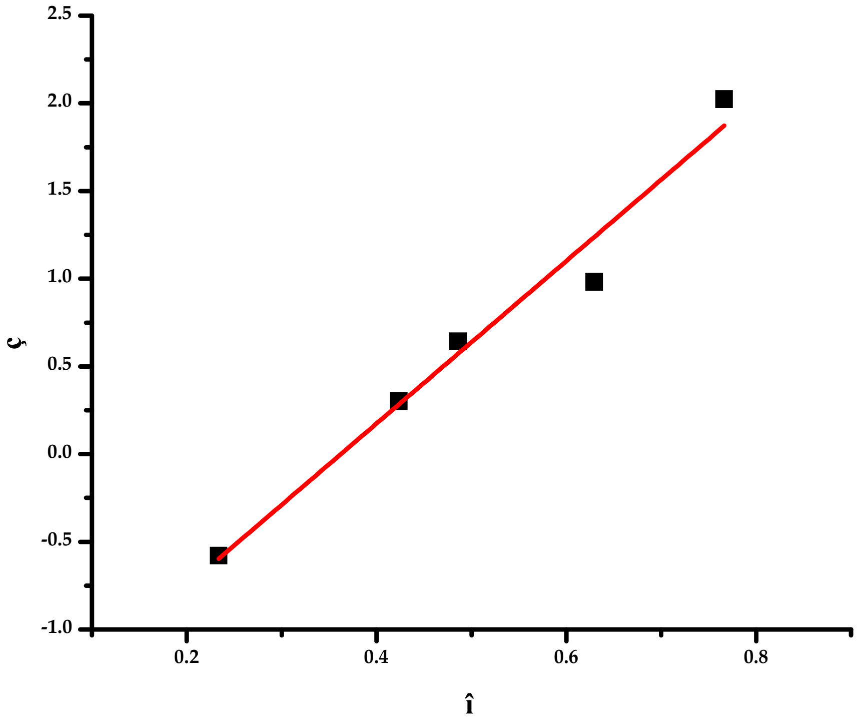

Figure S4. ext KT plot for the determination of the reactivity ratios for the PBVE-co-PCEVE

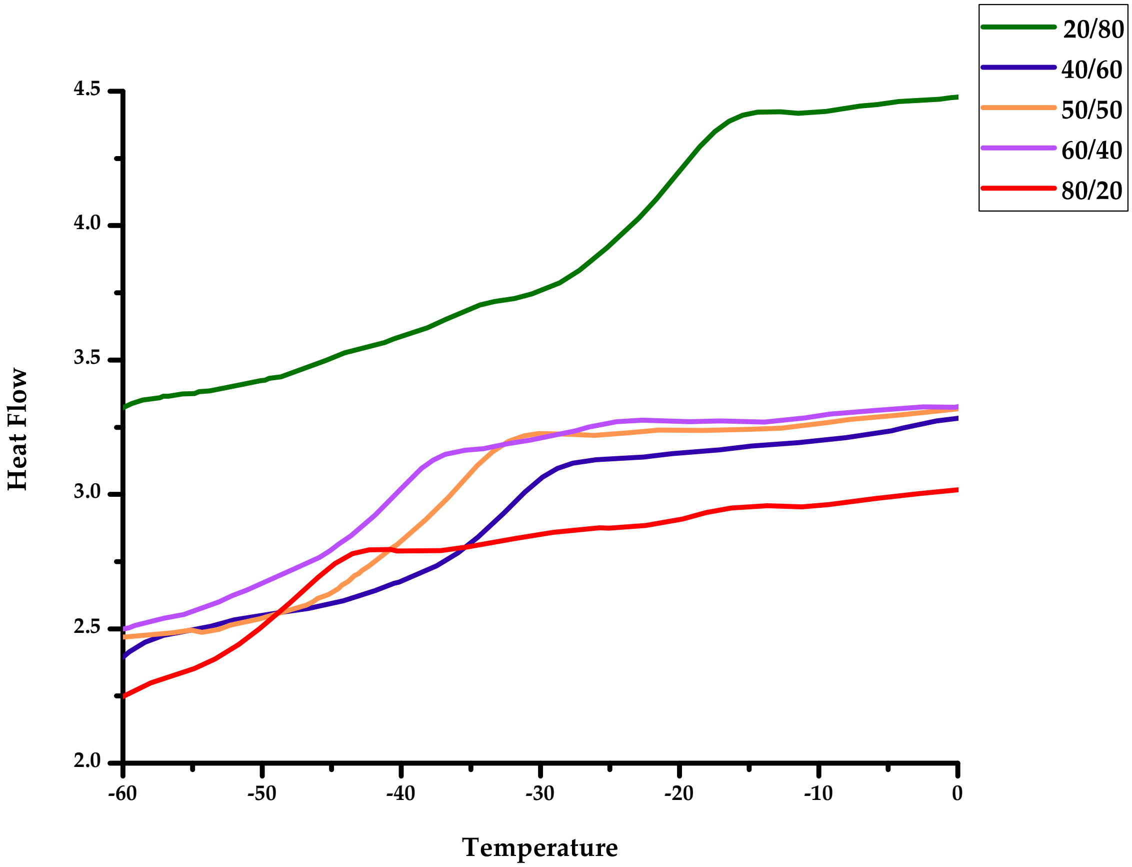

Figure S5. DSC thermograms for the statistical copolymers

Table S1. Copolymerization data for the synthesis of the statistical copolymers PBVE-co-PCEVE

SAMPLE	M_{BVE}	dM_{BVE}	M_{CEVE}	dM_{CEVE}	X	Y	G_m	H_m	G_m/H_m	$1/H_m$	η (KT)	ξ (KT)	η (ext KT)	ξ (ext KT)
**20/80**	0.2000	0.3100	0.8000	0.6900	0.2500	0.4493	-0.3065	0.1391	-2.2029	7.1884	-0.5038	0.2287	-0.5800	0.2336
**40/60**	0.4000	0.5900	0.6000	0.4100	0.6667	1.4390	0.2034	0.3089	0.6585	3.2378	0.2614	0.3970	0.3017	0.4234
**50/50**	0.5000	0.6800	0.5000	0.3200	1.0000	2.1250	0.5294	0.4706	1.1250	2.1250	0.5633	0.5007	0.6435	0.4860
**60/40**	0.6000	0.7600	0.4000	0.2400	1.5000	3.1667	1.0263	0.7105	1.4444	1.4074	0.8700	0.6023	0.9810	0.6294
**80/20**	0.8000	0.9100	0.2000	0.0900	4.0000	10.1111	3.6044	1.5824	2.2778	0.6319	1.7569	0.7713	2.0226	0.7664
